# Square beams for optimal tiling in TEM

**DOI:** 10.1101/2023.07.29.551095

**Published:** 2023-10-09

**Authors:** Eugene YD Chua, Lambertus M Alink, Mykhailo Kopylov, Jake Johnston, Fabian Eisenstein, Alex de Marco

**Affiliations:** 1- Simons Electron Microscopy Center, New York Structural Biology Center, New York, NY 10027; 2- Department of Physiology and Cellular Biophysics, Columbia University, New York, NY, USA; 3- Graduate School of Medicine, University of Tokyo, Tokyo, Japan.

**Keywords:** TEM, cryo-EM, square beam, TEM tiling

## Abstract

Imaging large fields of view at a high magnification requires tiling. Transmission electron microscopes typically have round beam profiles; therefore, tiling across a large area is either imperfect or results in uneven exposures, a problem on dose-sensitive samples. Here, we introduce a square electron beam that can be easily retrofitted in existing microscopes and demonstrate its application, showing it can tile nearly perfectly and deliver cryo-EM imaging with a resolution comparable to conventional setups.

In transmission electron microscopy (TEM) of dose-sensitive specimens such as vitrified biological material, pre-exposure of areas to the beam attenuates the attainable resolution (Baker & Rubinstein, 2010). High-resolution cryo-TEM typically comes at the cost of a reduced field of view; therefore, a balance between the pixel size and the sample imaged within its biological context is required. Since the illumination profile (which we call here “beam” or “beam profile” for simplicity) of TEMs is round, tiling across a large field of view encounters the circle packing problem, wherein circles cannot be perfectly tiled. Even with an ideal modern imaging setup with a fringe-free imaging (FFI) (Konings et al., 2019; Weis & Hagen, 2020) and a square sensor, the sensor will only capture ~69% of the area illuminated by the tightest possible round beam (Figure 1 and Supplementary Figure S1). The electron beam will damage the remaining illuminated but unimaged site, which will no longer contain high-resolution information when next imaged. This is a well-known limitation in montage tomography of vitrified specimens, and while data collection schemes that account for overlapping exposures exist (Peck et al., 2022; Yang et al., 2022), the illumination across multiple exposed areas remains non-uniform.

One solution to the problem of imperfect tiling with round beams is to use a square electron beam. Modern cryo-TEMs use Mueller-type sources where the emitter’s shape defines the electron beam shape, typically resulting in a circle. For TEM imaging in a 3-condenser system, the beam current (spot size) is selected by the C1 and C2 lenses, and the source beam width (beam convergence) is determined by changing the strength of the C2 and C3 lenses. The aperture between the C2 and C3 lenses becomes the beam-shaping aperture. When the electron beam cross-over above the C2 aperture is moved (by changing C1/C2 lenses), the beam current changes, whereas when the cross-over below the C2 is forced (by changing the C2/C3 lenses), the size of the beam changes. The post-C2-aperture beam takes on the shape of the aperture’s hole when the beam is spread more comprehensively than the aperture. For practical reasons linked to manufacturing and isotropic optical propagation, all cracks have round holes, creating round beams. In this work, we use a C2 aperture with a square hole to create a square electron beam profile. We demonstrate its utility on an FFI-capable TEM for near-perfect tiling in montage tomography and increased efficiency in data collection for single particle analysis with minimal loss of resolution.

Using a square C2 aperture, we successfully created a square beam (Figure 1C). First, we adjusted the beam width using the microscope intensity control such that the beam had the same size as the shortest dimension of the sensor. Then, the (post-objective) projection P2 lens was adjusted to rotate the beam square onto the sensor (Figure 1C). Aligning the beam with the sensor ensures that the sensor images the entire sample area exposed to the beam. Since changes in the P2 lens strength changed the image’s rotation, magnification, and defocus, calibrations for pixel size, image shift, and eucentric focus had to be redone. The flux on the sensor can be adjusted with spot size, and the beam intensity distribution across the illuminated area can be measured to ensure uniform exposures (**Error! Reference source not found**.). The unique P2 lens state can be stored as a unique magnification entry and added as a separate registry key.

With a square beam, it became possible to tile with minimal overlap to exhaustively image a large field of view. This is especially important for *in situ* tomography, where it is often helpful to image large contiguous areas of a specimen, such as a lamella, at high resolution. Acquisition targets can be set along the tilt axis to overlap minimally and, therefore, reduce any areas on the sample that are exposed to the electron beam in more than one acquisition target. To maximize the acquisition area while avoiding the sample overexposure in the direction perpendicular to the tilt axis, a new data acquisition scheme was developed, where the beam-image shift is independent of the sample tilt. This scheme was implemented in PACE-tomo (Eisenstein et al., 2023), and the beam shifts were set to be equivalent to one image Y-axis in nm (Figure 1D–I and Supplementary Videos). The beam overlap was maintained identically throughout the tilt series (shows the difference between conventional tiled at 0 deg versus camera-based offset). After data acquisition, a montage for each stage tilt can be stitched to produce a sizeable field-of-view image, which is then aligned across the tilt series and reconstructed.

In single particle data acquisition, a square beam significantly increases throughput. Using common supports such as UltrAuFoil R1.2/1.3 grids, a square beam could image up to 5 targets per hole (85 targets per stage movement) versus 2 targets per hole for a round beam (Supplementary Figure S4). This increased the data collection rate by nearly 2-fold, from ~158 exposures per hour with the round beam to ~291 exposures per hour with the square beam.

While we observed normal behavior during microscope alignment and coma correction (Supplementary Figure S5), we consistently obtained a slightly worse reconstruction B-factor for the square beam. However, with enough particles, the reconstruction still went to Nyquist (Figure 2). With smaller particle sets, we consistently observed a slight loss in reconstruction resolution (~0.1 Å) with the square beam compared to the round beam (Supplementary Figure S8). This is most likely linked to the lack of circular symmetry in the phase profile of the beam diffraction from the square aperture. A potential solution is to use larger apertures to lower the diffraction angle and maintain a more uniform phase profile at the sample plane.

The most notable change from the round to the square beam is that the spherical aberration fit increases from 2.9 to 3.1 mm during homogeneous refinement. The increased spherical aberration happens with the detuning of the P2 lens – the spherical aberration did not change with square aperture data collected without P2 lens detuning.

Considering the change in the tuning of the P2 lens, we tested the potential changes in microscope performance. The magnification was not subject to any measurable distortion (Supplementary Table S1) (Grant & Grigorieff, 2015). Further, Young’s Fringes experiment showed that the microscope performance remained within the manufacturer’s specification as the information transmittance reached 0.14 nm on gold cross-grating (Supplementary Figure S9).

Non-circular and square beams have been developed for beam-shaping in electron beam lithography, laser micromachining, and medical laser applications. We use a square beam for nearly perfect cryo-EM and cryo-ET montage tiling. Other non-round beams, such as rectangles and hexagons, which are also optimal profiles for tiling, can be used (Brown et al., 2023). We note that it is possible to create a rectangular beam by stigmating a square beam; however, this introduces unwanted aberrations into the beam. Furthermore, matching the beam’s and detector’s shapes prevents minor data processing complications associated with having unilluminated sensor areas.

Among optimizations, the alignment of the aperture orientation with respect to the sensor is critical to ensure optimal overlap between the sensor and the illuminated area. As an alternative to rotating the beam with the P2 lens, we envision that the aperture can be mechanically rotated through a redesign of the aperture strip: a worm wheel gear can be installed to physically rotate the square aperture while it is in the liner tube under vacuum during illumination. For this work, we propose a no-cost solution that involves adjusting the P2 lens’ current to induce a rotation of the projected image plane. This introduces changes in the optical system, affecting the image’s magnification, defocus, and rotation, requiring recalibration of the pixel size, eucentric focus, and image-shift matrices.

## Supplementary Material

1

## Figures and Tables

**Figure 1.**
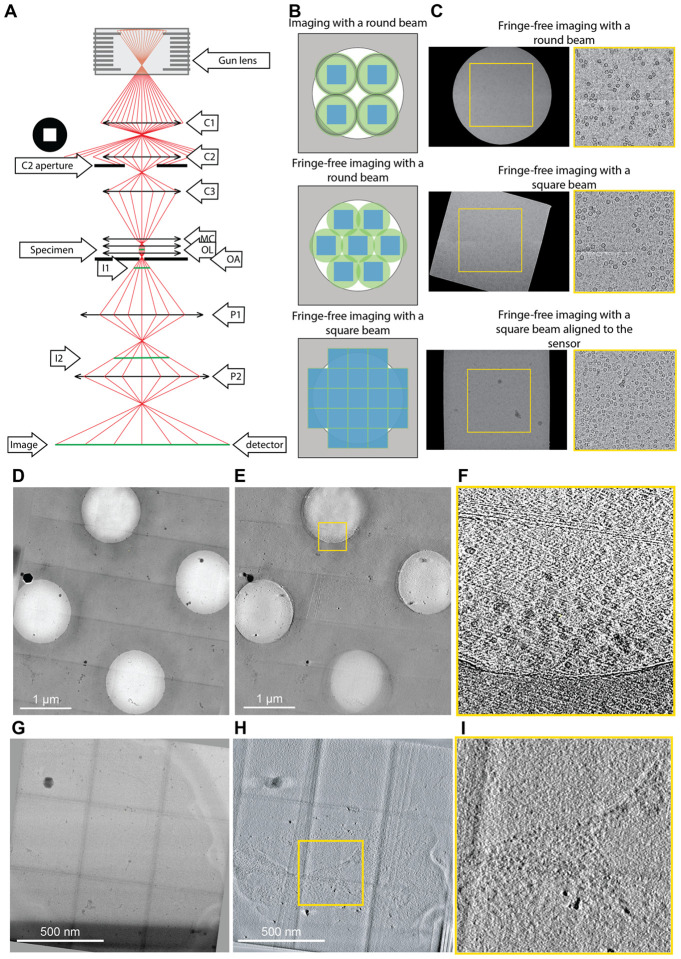
(A) Ray diagram of electrons passing through the column of a TEM. The square aperture is placed at the “C2 aperture” position. C1/2 - condenser lens 1/2, OL - objective lenses, OA - objective aperture, MC - minicondenser, I1/2 - intermediate image plane 1/2, P1/2 - projection lens 1/2. (B) Examples of imaging a specimen (white circle) with different TEM beam setups. The areas on the specimen illuminated by the electron beam are shown in green circles (round beam) or green squares (square beam); the illuminated areas are top image, black rings) in non-fringe-free TEM setups, the beam must be spread out so the fringes do not fall on the sensor. (C) Example micrographs from an FFI-enabled TEM, acquired with a round beam (top), square beam (middle), and a square beam aligned to the sensor by adjusting the projection lens (bottom). (D) Example of PACE-tomo tiled imaging with the square beam. We collected a 5×5 butt-joint tile set on holey carbon grids with apoferritin. (E) The resulting tomogram reconstructed from the data shown in D. (F) High magnification crop of the joint between two tiles from panel E. In the upper section, imperfections in the stitching are visible, as no alignment or interpolation was performed when stitching. However, the apoferritin particles are clearly visible. (G) PACE-tomo tiled imaging with the square beam on yeast lamellae. Here, we collected a 3×3 butt-joint tile set. (H) The resulting tomogram reconstructed from the data shown in G. (I) High magnification crop of a region of the tomogram.

**Figure 2.**
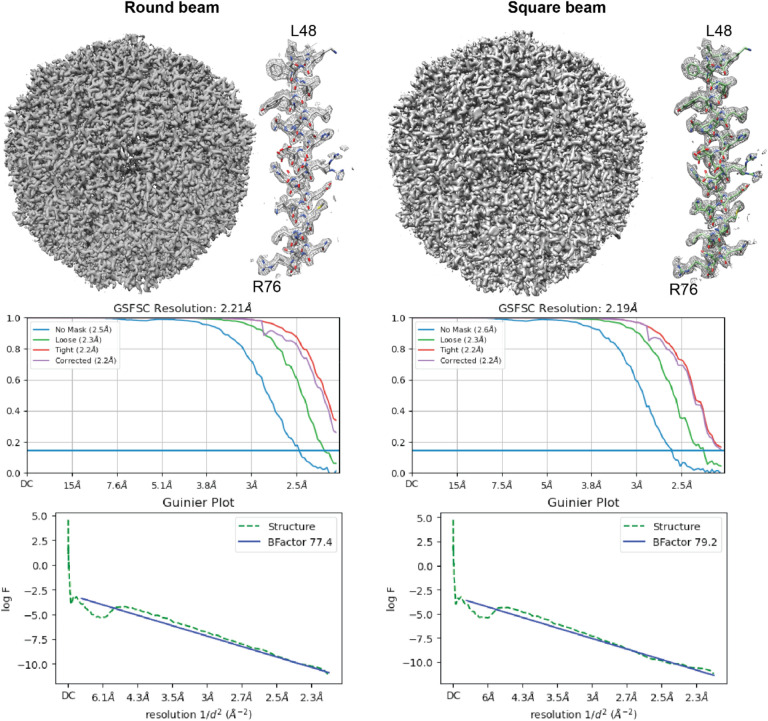
Single particle reconstructions of apoferritin with data collected with a round (left) or a square (right) beam. In both cases, with 120,000 particles, the reconstructions can achieve Nyquist resolution.

## Data Availability

Data will be made freely available upon request.
