# Supplementary material for: Square beams for optimal tiling in TEM
Source: bioRxiv. 2023 Oct 9:2023.07.29.551095. Preprint. [Version 2] doi: 10.1101/2023.07.29.551095 (PMC10592621; doi:10.1101/2023.07.29.551095)
Supplement: 1 [file NIHPP2023.07.29.551095V2-supplement-1.pdf]

bioRxiv preprint doi: https://doi.org/10.1101/2023.07.29.551095; this version posted October 9, 2023. The copyright holder for this preprint (which was not certified by peer review) is the author/funder, who has granted bioRxiv a license to display the preprint in perpetuity. It is made available under aCC-BY 4.0 International license.

Supplementary material

Methods

Square apertures were purchased from Agar Scientific (product numbers AGAS3005P and AGAS3005P). The apertures are made of platinum, with a diameter of 3.04 mm, a thickness of 0.25 mm, and a square hole of 50 or 100 µm. A 50-micron aperture has been installed into the C2 aperture holder of the microscope. Before its installation into the microscope, the square aperture should be plasma cleaned to remove any impurities, then maintained in a sealed container for several days to allow the charge to dissipate, allowing an easier insertion into the aperture strip.

The optics of the electron microscope consists roughly of three sections: the beam forming condenser section where the square aperture is installed, the objective section with the specimen, and the magnifying projection lens section. See also Figure 1A. The square aperture is located at the condenser section, defining the square beam. To align the square beam towards the camera, we tuned the P2 projection lens. The condenser and objective lens settings were not touched to keep the parallelism of the beam intact going through the objective system. With the electron microscope set to diffraction mode, on de-tuning of the condenser lens, the diffraction rings showed blurring, indicating loss of parallelism of the beam in the objective lens. Detuning of the projection system left the parallelism intact. This was expected since the projection system is below the objective lens. Note that tuning the projection lens will affect the image's magnification, rotation, and defocus.

The strength of the P2 lens can be checked in the user interface system status overview (Supplementary Figure S6). The square aperture can be inserted using the standard OCX in the user interface. The square C2 aperture has the same form factor as the 'standard' round C2 aperture. Replacement of the C2 aperture, aligning the aperture laterally, and tuning the P2 lens can be done by the equipment supplier service engineer using the standard software and wizards. A screenshot of the aperture wizard is given in Supplementary Figure S7. After the adjustments, eucentric focus calibration, pixel size, and image shift calibrations must be performed in the SerialEM (Mastronarde, 2005).

The same protein sample was used for apoferritin tomography and single particle analysis prepared as described below, with the only difference found in the support (carbon vs gold). Data were acquired using PACE-tomo scripts (Eisenstein et al., 2023) incorporated into SerialEM 4.1 beta 13 (Mastronarde, 2005) with a pixel size of 2.12 Å/px, exposure dose of 3.4 $e^-/Å^2$ per tilt, and -45° to 45° tilt range for a total of 31 tilts and total dose of 105 $e^-/Å^2$ per tilt series. Imaging was done as 5x5 patches. Saccharomyces cerevisiae standard yeast test sample for lamella tomography was prepared following the Waffle method protocol (Kelley et al., 2022; Klykov et al., 2022). Data were acquired using PACE-tomo scripts incorporated into SerialEM 4.1 beta 13 with a pixel size of 2.12 Å/px, exposure dose of 2.55 e^-/ $Å^{2,}$ and a total dose of 76.5 e^-/ $Å^2$. Imaging was done as 3x3 patches to encompass the whole yeast cell in the field of view.

bioRxiv preprint doi: https://doi.org/10.1101/2023.07.29.551095; this version posted October 9, 2023. The copyright holder for this preprint (which was not certified by peer review) is the author/funder, who has granted bioRxiv a license to display the preprint in perpetuity. It is made available under aCC-BY 4.0 International license.

For the apoferritin dataset, the acquired tilt series were first motion corrected with Warp (Tegunov & Cramer, 2019), and tilt series were aligned and reconstructed with AreTomo (Zheng et al., 2022) at bin6 and used without additional processing for further analysis. For the yeast lamella dataset, the tilt series were motion-corrected with Warp (Tegunov & Cramer, 2019) and aligned using AreTomo (Zheng et al., 2022). Tomogram reconstruction was performed using Tomo3D (Agulleiro & Fernandez, 2015), and the tomogram was deconvolved IsoNet (Liu et al., 2022) to enhance contrast. Stitching for both the apoferritin and yeast lamella datasets was done automatically with custom Python scripts, where the tiles were stitched together using the image shift locations obtained from the metadata.

For single particle analysis, UltrAuFoil R1.2/1.3 300 mesh Au grids were hydrophilized with a mixture of Ar and O_2 gas (26.3:8.7 ratio) at 15 W for 7 seconds in a Solarus Model 950 Advanced Plasma System (Gatan). 3 µl of 8 mg/ml mouse apoferritin was pipetted onto each grid, blotted for 3-5 seconds in a Vitrobot at 20°C and 100% relative humidity, then vitrified in liquid ethane. The P2 projection lens was detuned to rotate the square beam square onto the sensor, resulting in changes in the image's magnification, rotation, and defocus. Eucentric focus needed to be reset on the microscope by adjusting the objective lens, then beam and image shift and scale rotation calibrations needed to be redone in Leginon (Cheng et al., 2021; Suloway et al., 2005) prior to data collection. Pixel size calibration was done in SerialEM on a standard cross-grating replica grid. Energy filter alignments were done per standard protocol, with the entire sensor illuminated. Objective lens astigmatism and coma correction were performed using Sherpa, with the full sensor illuminated. Single particle data were collected using Leginon with either a 100 µm round C2 aperture or a 50 µm square C2 aperture. Data were collected at a pixel size of ~1.08 Å/pixel, a flux of ~30 e/px/s for 2 seconds, equaling a total dose of ~51 e/Å^2, with a nominal defocus range of -0.5 µm to -2.0 µm. The square beam was condensed to match the size of the sensor to maximize the data acquisition area, and in the control experiment, a round beam was used with its intensity set to match the flux of the square beam on the sensor. Data were collected using a beam-image shift (Cheng et al., 2018).

For single particle data processing, square and round beam data was first motion corrected with patches in cryoSPARC v4.2.1 (Punjani et al., 2017). Full micrographs were then patch CTF estimated, and particles were picked using apoferritin templates. Particles were extracted with a box size of 280 pixels, 2D classified, and 120,000 particles randomly selected for homogeneous refinement. To exclude the unilluminated areas of the sensor because of the condensed square beam, a central square region of the motion-corrected micrographs was cropped out with the IMOD (Mastronarde & Held, 2017) command "trimvol". As a control, the same central square region was cropped from both square and round aperture data. Cropped micrographs were then re-imported into cryoSPARC, and the same processing workflow continued as above. Reconstructions from full and cropped micrographs from square and round aperture data were compared.

bioRxiv preprint doi: https://doi.org/10.1101/2023.07.29.551095; this version posted October 9, 2023. The copyright holder for this preprint (which was not certified by peer review) is the author/funder, who has granted bioRxiv a license to display the preprint in perpetuity. It is made available under aCC-BY 4.0 International license.

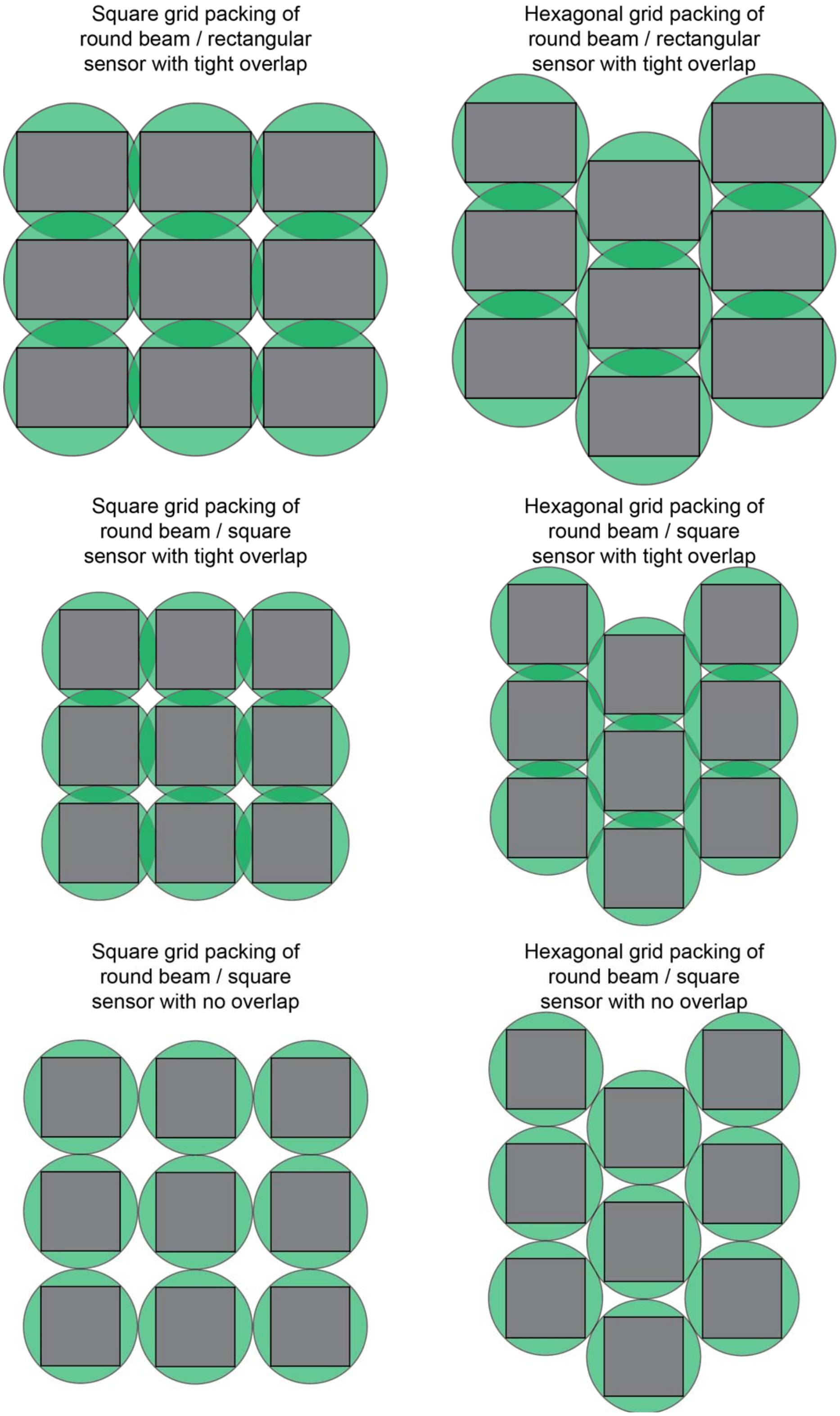

Supplementary Figure S1. Examples of packing circular beams (green) and square or rectangular sensors (grey) highlighting the gap between the exposed and imaged areas.

bioRxiv preprint doi: https://doi.org/10.1101/2023.07.29.551095; this version posted October 9, 2023. The copyright holder for this preprint (which was not certified by peer review) is the author/funder, who has granted bioRxiv a license to display the preprint in perpetuity. It is made available under aCC-BY 4.0 International license.

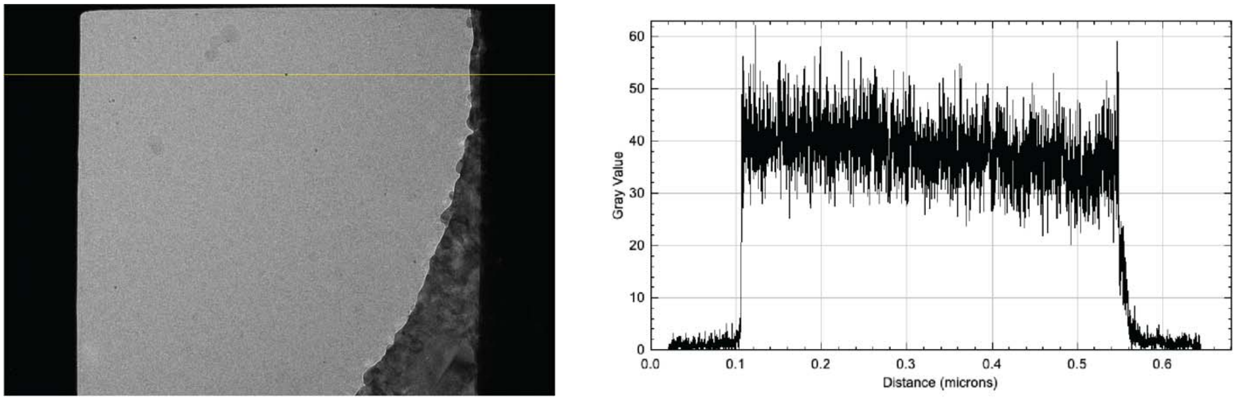

Supplementary Figure S2. Example micrograph taken with a square beam (left), showing a pixel intensity profile (right) for the pixels along the yellow line. Pixel intensity profile was obtained with ImageJ (Schneider et al., 2012).

bioRxiv preprint doi: https://doi.org/10.1101/2023.07.29.551095; this version posted October 9, 2023. The copyright holder for this preprint (which was not certified by peer review) is the author/funder, who has granted bioRxiv a license to display the preprint in perpetuity. It is made available under aCC-BY 4.0 International license.

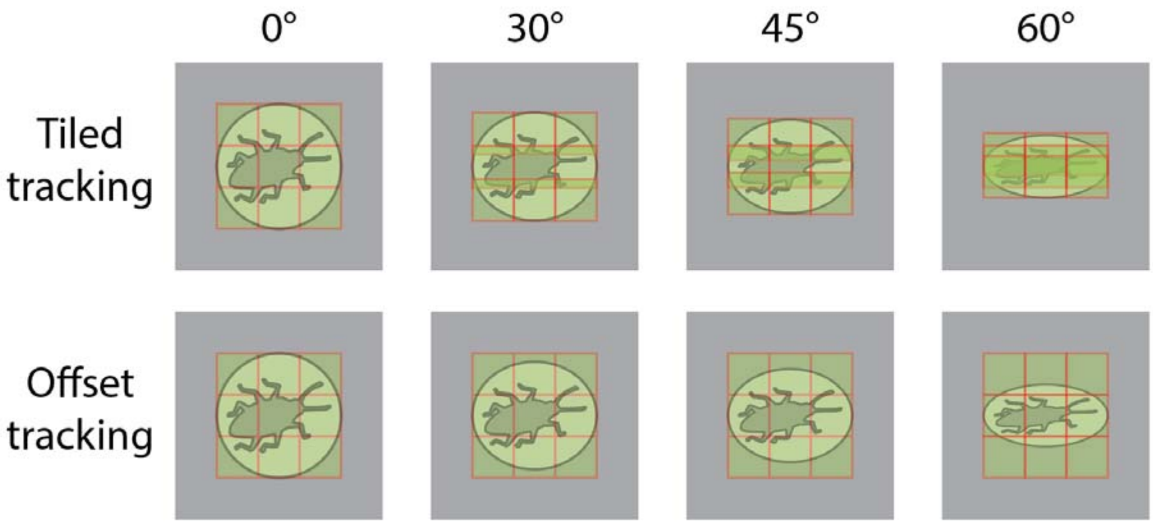

Supplementary Figure S3. Tomography data acquisition schemes with the square beam and the effect of sample tilt on beam overlap when using a fixed beam offset (tiled tracking). We implemented an acquisition scheme where the position of the tile is determined by the Y-axis length of the image in nm (offset tracking).

bioRxiv preprint doi: https://doi.org/10.1101/2023.07.29.551095; this version posted October 9, 2023. The copyright holder for this preprint (which was not certified by peer review) is the author/funder, who has granted bioRxiv a license to display the preprint in perpetuity. It is made available under aCC-BY 4.0 International license.

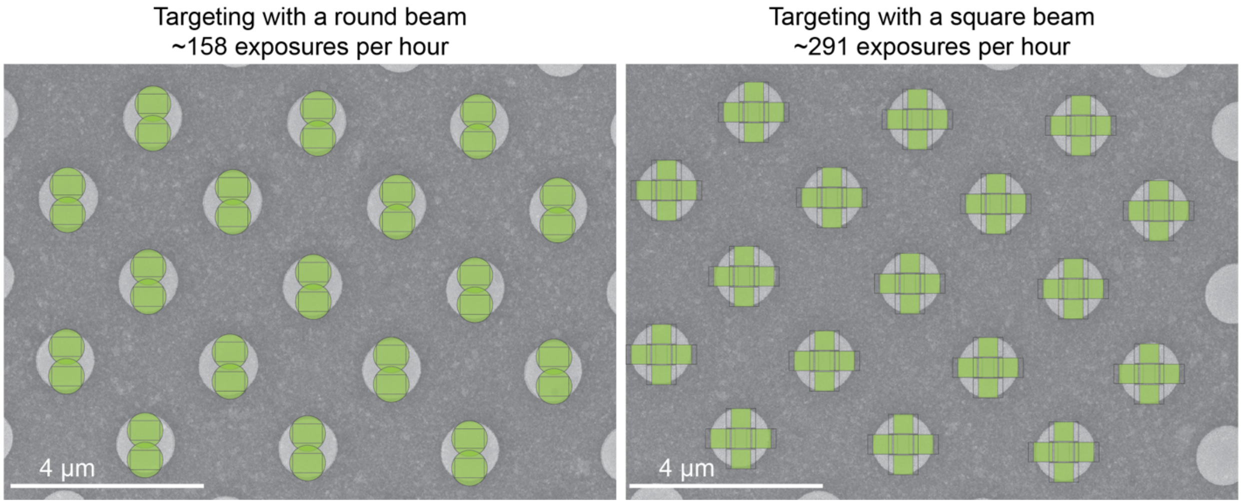

Supplementary Figure S4. When using a round beam and fringe-free imaging, two acquisition targets can be acquired for each 1.2 µm hole (left). In this field of view, up to 34 acquisition images can be taken per stage movement. When using a square beam with perfect tiling, five acquisition targets can be acquired for each 1.2 µm hole (right), increasing the number of images to 85 per stage movement.

bioRxiv preprint doi: https://doi.org/10.1101/2023.07.29.551095; this version posted October 9, 2023. The copyright holder for this preprint (which was not certified by peer review) is the author/funder, who has granted bioRxiv a license to display the preprint in perpetuity. It is made available under aCC-BY 4.0 International license.

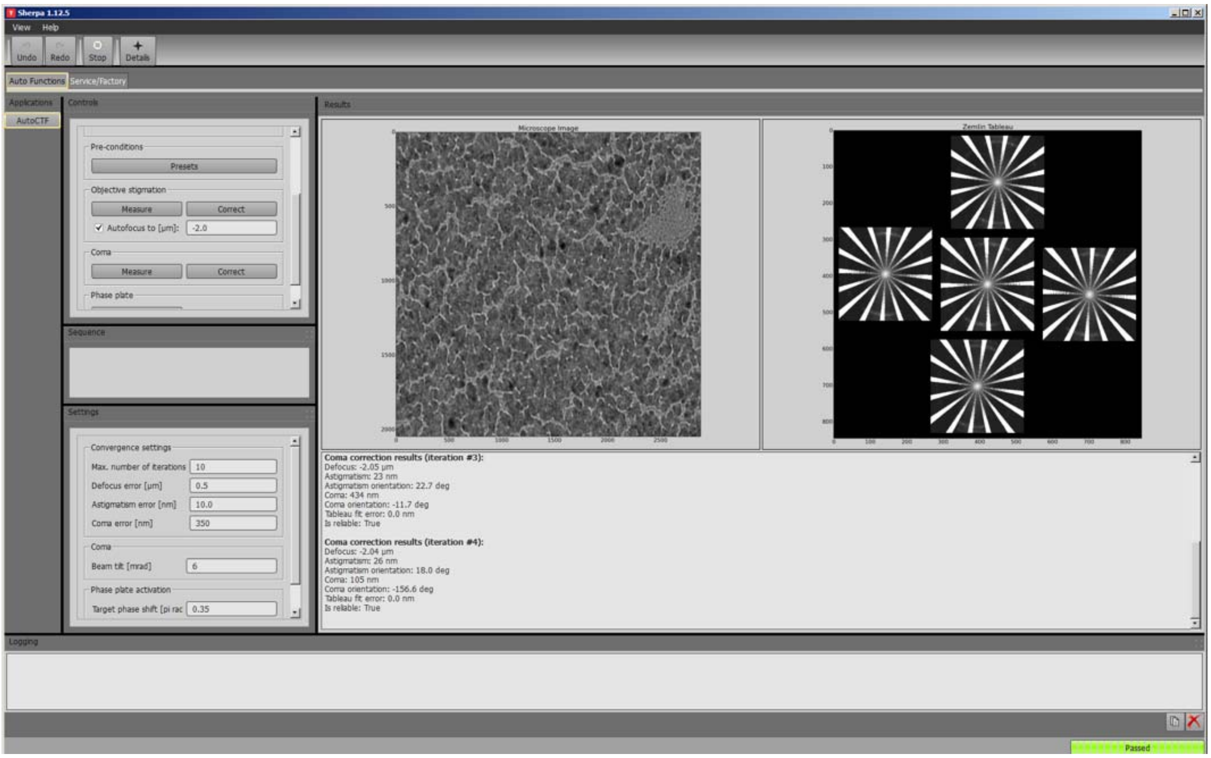

Supplementary Figure S5. Automated coma correction while using a square beam shows it is possible to achieve acceptable levels of coma, directly comparable to using round illumination.

bioRxiv preprint doi: https://doi.org/10.1101/2023.07.29.551095; this version posted October 9, 2023. The copyright holder for this preprint (which was not certified by peer review) is the author/funder, who has granted bioRxiv a license to display the preprint in perpetuity. It is made available under aCC-BY 4.0 International license.

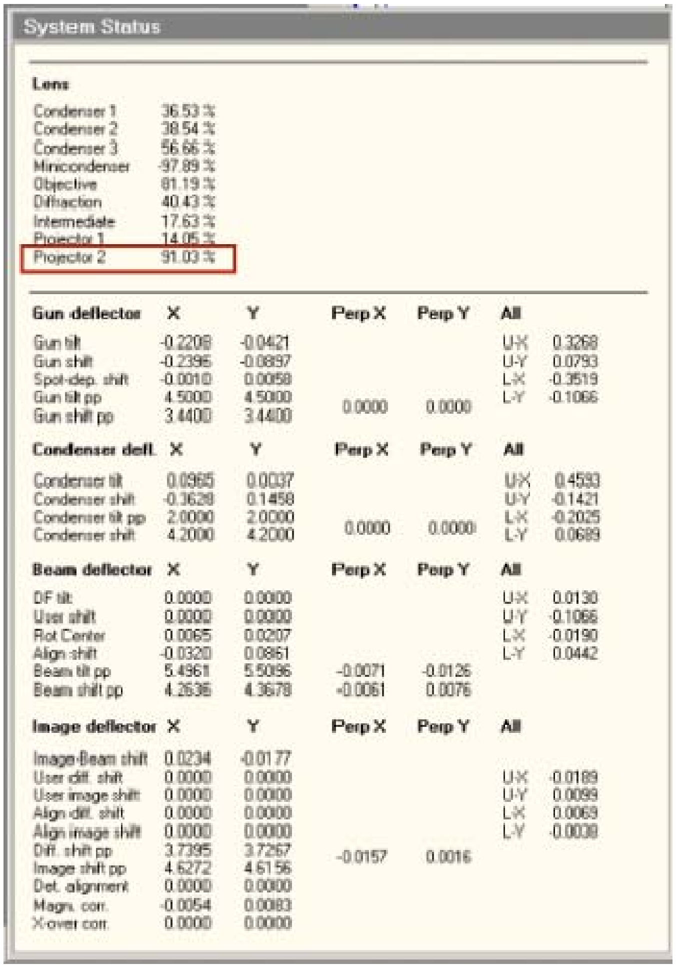

Supplementary Figure S6. System status overview in the user interface showing the strength of the P2 lens.

bioRxiv preprint doi: https://doi.org/10.1101/2023.07.29.551095; this version posted October 9, 2023. The copyright holder for this preprint (which was not certified by peer review) is the author/funder, who has granted bioRxiv a license to display the preprint in perpetuity. It is made available under aCC-BY 4.0 International license.

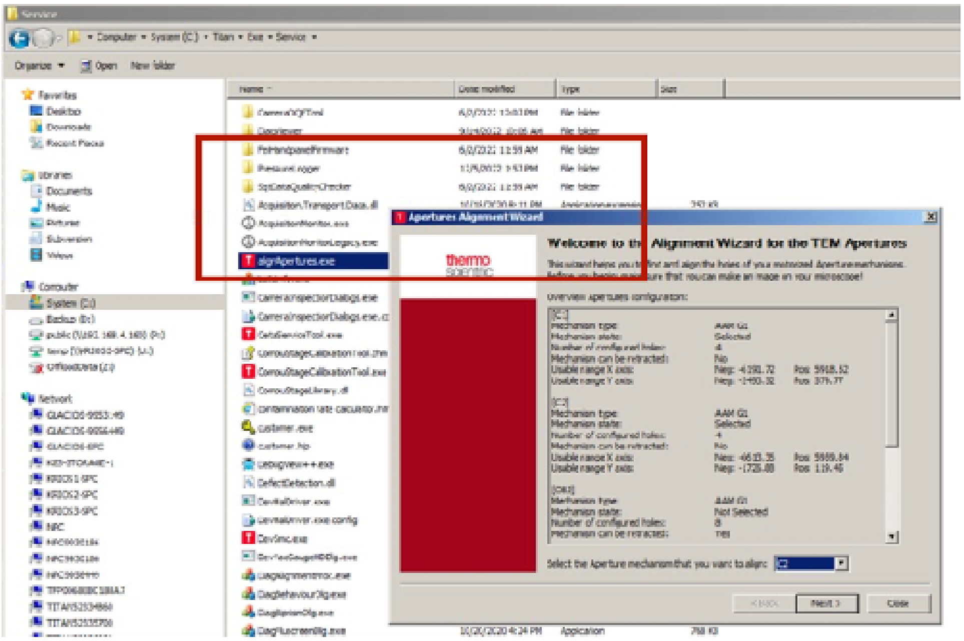

Supplementary Figure S7. *Aperture alignment wizard that allows adjusting the C2 aperture.*

bioRxiv preprint doi: https://doi.org/10.1101/2023.07.29.551095; this version posted October 9, 2023. The copyright holder for this preprint (which was not certified by peer review) is the author/funder, who has granted bioRxiv a license to display the preprint in perpetuity. It is made available under aCC-BY 4.0 International license.

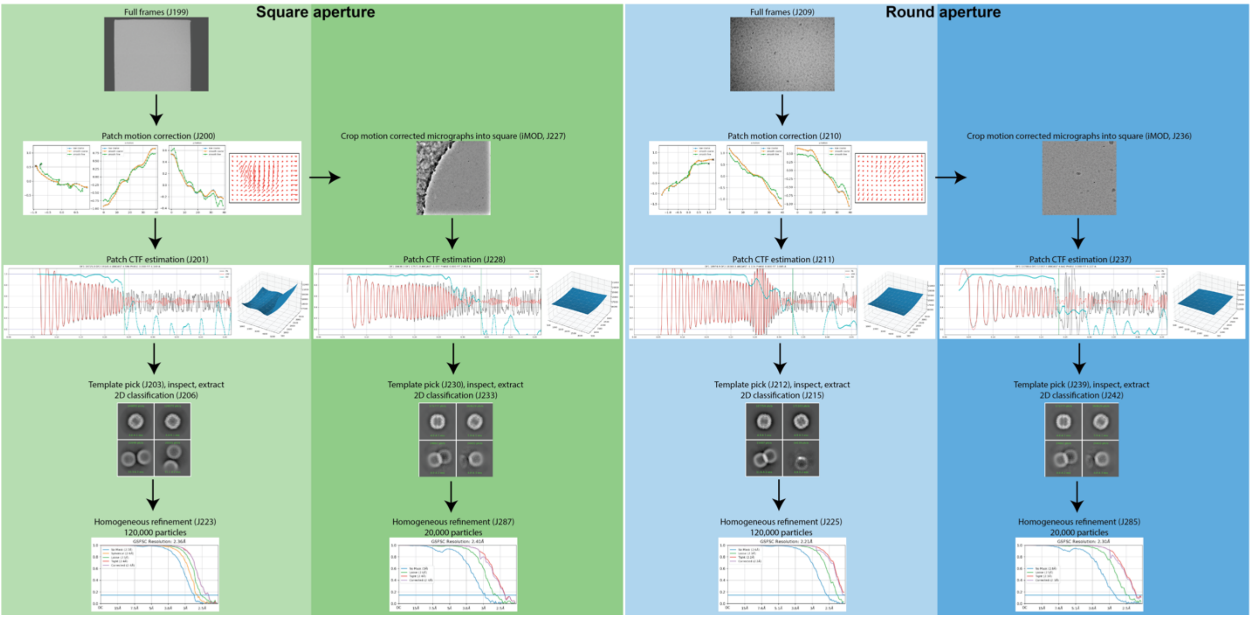

Supplementary Figure S8. Single particle data processing workflow for the square (green) and round (blue) apertures. For each aperture type, the processing workflow uses the full frames on the left, and the processing workflow with cropped micrographs (to exclude the unilluminated areas of the sensor) on the right.

bioRxiv preprint doi: https://doi.org/10.1101/2023.07.29.551095; this version posted October 9, 2023. The copyright holder for this preprint (which was not certified by peer review) is the author/funder, who has granted bioRxiv a license to display the preprint in perpetuity. It is made available under aCC-BY 4.0 International license.

354

C2 aperture	P2 lens rotated?	Magnification	Distortion angle (degrees)	Major axis scale factor	Minor axis scale factor	Distortion (%)
Round, 100 µm	No	81,000	8.6	1.002	0.998	0.45
Square, 50 µm	No	81,000	10.5	1.002	0.998	0.45
Round, 100 µm	Yes	~81,000	27.6	1.002	0.998	0.35

355 **Supplementary Table S1.** Anisotropic magnification distortion. We performed a common measurement of the magnification
356 along all axes using the program described by Grant and Grigorieff (Grant & Grigorieff, 2015). We tested two different apertures
357 and imaged with the conventional (P2 lens rotated? = No) and new P2 lens tuning (P2 lens rotated? = Yes). All the conditions
358 show the same distortion.

359

bioRxiv preprint doi: https://doi.org/10.1101/2023.07.29.551095; this version posted October 9, 2023. The copyright holder for this preprint (which was not certified by peer review) is the author/funder, who has granted bioRxiv a license to display the preprint in perpetuity. It is made available under aCC-BY 4.0 International license.

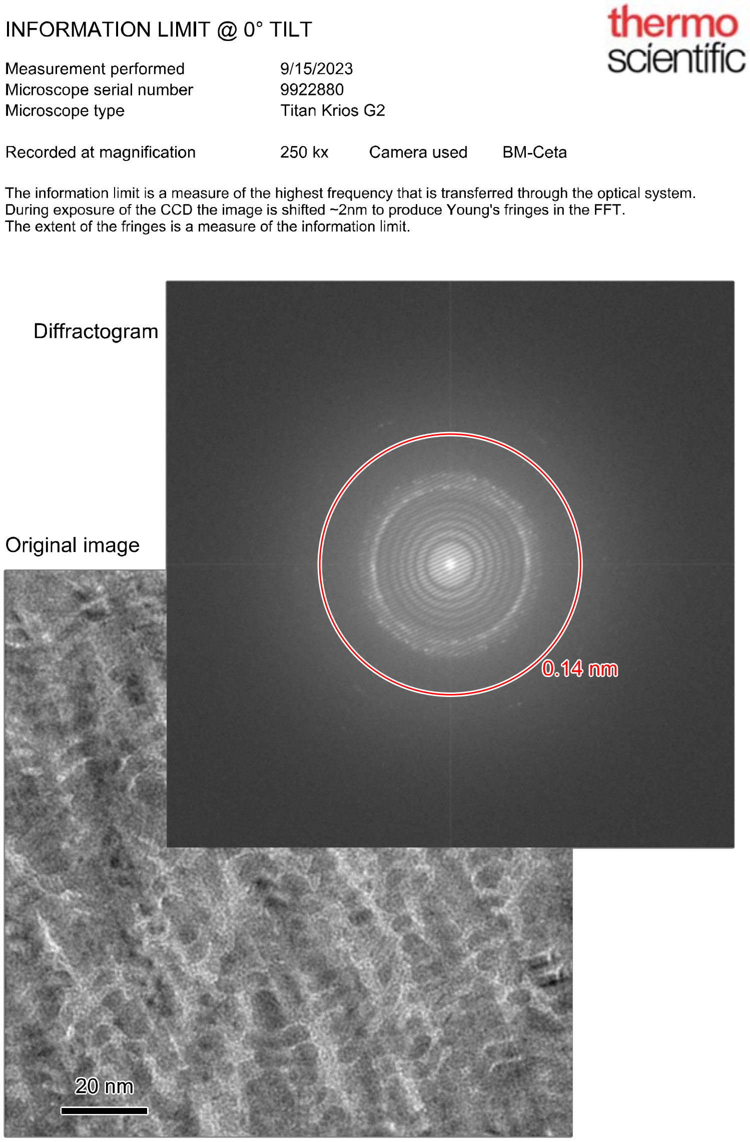

Supplementary Figure S9. Screenshot of the standard output from the Young's fringes test performed by Thermo Fisher service. This test shows the transmittance limit of the microscope, and in this case, it demonstrates that the use of a square aperture and a differently tuned projection system does not impact on microscope performance.
